# Scleral fixation using a hydrophilic four-haptic lens and polytetrafluoroethylene suture

**DOI:** 10.1038/s41598-021-95428-2

**Published:** 2021-08-04

**Authors:** Natacha B. Junqueira, Leandro J. Chaves, Omero Poli-Neto, Ingrid U. Scott, Rodrigo Jorge

**Affiliations:** 1 Department of Ophthalmology, Ribeirão Preto Medical School, University of São Paulo, Avenida Bandeirantes, 3900, Ribeirão Preto, SP 14049-900 Brazil; 2 Federal University of Mato Grosso Do Sul, Três Lagoas, MS Brazil; 3 Departments of Ophthalmology and Public Health Sciences, Penn State College of Medicine, Hershey, PA USA

**Keywords:** Clinical trial design, Surgery

## Abstract

To assess the safety of scleral fixation using the Akreos AO60 intraocular lens (IOL) and Gore-Tex suture. Prospective evaluation of 20 patients who underwent scleral fixation of an Akreos AO60 with Gore-Tex. Patients presenting with aphakia or dislocated IOL without capsular support were enrolled in the study. Main outcome measures included visual acuity, endothelial cell density, and postoperative complications over 6 months of follow-up. Mean ± standard deviation (SD) uncorrected logMAR visual acuity improved from 1.92 ± 0.23 (20/1600 Snellen equivalent) preoperatively to 0.80 ± 0.56 (20/125) at 6 months postoperatively (*p* < 0.001). Mean ± SD best-corrected visual acuity (BCVA) logMAR was 0.43 ± 0.23 preoperatively and 0.37 ± 0.24 (20/50) at 3–6 months postoperatively (*p* = 0.312). The mean ± SD endothelial cell density was 1740.50 ± 522.92 cells/mm^2^ and 1187.19 ± 493.00 cells/mm^2^ (*p* < 0.001) pre and postoperatively, respectively. Mean ± SD postoperative spherical equivalent was − 1.12 ± 1.50D. Postoperative complications included exposure of suture in 40% of the patients, hypotony in 15%, ocular hypertension in 10%, transient vitreous hemorrhage in 10%, retinal detachment in 5%, and transient lens opacification in 5%. Scleral fixation with an Akreos AO60 and Gore-Tex appears generally safe. However, given the high incidence of suture erosion observed, the use of scleral flaps or rotating and burying the knots is recommended in order to reduce the risk of this complication.

## Introduction

In the context of inadequate capsular support, surgical options for intraocular lens (IOL) implantation include insertion of an anterior chamber IOL (ACIOL), iris fixation of an ACIOL, iris fixation of a posterior chamber IOL (PCIOL), and scleral fixation (with or without suture) of a PCIOL.

Technique selection is often influenced by patient age, anatomical factors (e.g*.*, previous trauma with loss of iris tissue), other ocular comorbidities (such as glaucoma), and surgeon preference.

Older ACIOLs have been associated with higher rates of ocular hypertension, loss of endothelial cells, pupillary block, cystoid macular edema, and uveitis-glaucoma-hyphema syndrome, when compared to PCIOLs^1,2^. Further, with PCIOLs, the position of the lens is closer to the natural lens location, probably offering refractive benefits^1^.

Iris fixation has been associated with higher rates of iris atrophy, pigmentary dispersion syndrome, uveitis, and pseudophacodonesis when compared to scleral fixation^3^. However, the scleral fixation technique is also not without complications. Retinal detachment, IOL inclination, vitreous and suprachoroidal hemorrhage, endophthalmitis, and suture erosion/breakage have all been reported and can result in permanent loss of sight^4,5^.

Uncertainties regarding the erosion and breaking of thread sutured to the sclera and the PCIOL have received special attention. Concerns about the half-life of commonly used polypropylene thread have been raised. Some scleral fixation techniques without suture have been suggested^6,7^, as well as alternative suture materials, such as Gore-Tex or larger diameter polypropylene (9-0)^4,5,8^, to minimize the risk of suture-related complications.

In 2014, Khan et al.^1^ described a modified technique for scleral fixation at two points of a CZ70BD IOL (Alcon, Fort Worth, TX, USA) using Gore-Tex suture, a non-absorbable monofilament polytetrafluoroethylene (PTFE) thread. With increased experience, the technique proposed by Khan et al.^1^ has undergone modifications. The main adjustment was the use of an Akreos AO60 IOL with a four-haptic design that enables excellent centralization and stabilization of the IOL through four-point fixation^9^. Recent studies using this technique have been published, although none were prospective^5,7,9–13^.

The objective of the current study was to evaluate the safety of Akreos AO60 scleral fixation using Gore-Tex suture, in the absence of capsular support, associated with pars plana vitrectomy (PPV), when necessary.

## Methods

### Study design

This prospective single-center study adhered to the principles of the Declaration of Helsinki and was approved by the local research ethics committee of the Ribeirão Preto Medical School of the University of São Paulo (reference number: 03907418.5.0000.5440). Trial registration number: RBR-6qvmjs (09/02/2020).

All aphakic patients, and those with dislocated or subluxed IOLs without capsular support, treated at the Clinical Hospital of Ribeirão Preto Medical School between February and May 2019 were invited to participate in this study. Written informed consent was obtained from all participants.

### Study population

Inclusion criteria were as follows: patients aged ≥ 18 years with aphakia or decentered/dislocated IOL; absence of reliable capsular support; and best-corrected visual acuity (BCVA) at least 5 letters better than uncorrected visual acuity.

Exclusion criteria included the presence of capsular support that would render it possible to implant an IOL without scleral fixation, ocular comorbidities that could be aggravated by surgery, such as advanced glaucoma and corneal disease, endothelial cell count density less than 1000/mm^2^, and the presence of systemic comorbidities contraindicating the surgical procedure.

### Baseline and follow-up evaluations

Patients underwent comprehensive ophthalmological evaluations at baseline, one day after surgery, one week after surgery, and after 1, 2, 3, and 6 months post-surgery. The baseline assessment included uncorrected and BCVA measurements, applanation tonometry, slit-lamp biomicroscopic examination, indirect fundus examination, specular microscopy and pachymetry (ICONAN - Konan Specular Microscope Sp-5500, Irvine, CA, USA), and immersion biometry.

Preoperative lens calculations were carried out using immersion ultrasonography (Alcon OcuScanRxP, Fort Worth, TX, USA). The SRK-T (axial length greater than 26 mm), Hoffer-Q (axial length less than 22 mm), or Holladay 1 formula was used, depending on the obtained value. The calculation was made for the implantation of an IOL in the topography of the capsular bag. The target postoperative refraction average was − 0.40 diopter spherical equivalent.

At all visits, uncorrected visual acuity and intraocular pressure (IOP) were measured, and slit-lamp biomicroscopic and indirect fundus examinations were performed. Optical coherence tomography (SD-OCT; Heidelberg Engineering, Germany) was performed in patients with a postoperative BCVA worse than 20/40 and who were believed to have an epiretinal membrane (ERM) or cystoid macular edema (CME) based on fundus biomicroscopy. Refraction, specular microscopy and pachymetry were performed between 3 and 6 months after surgery, at which time BCVA was measured.

Snellen visual acuities were converted into mean logMAR equivalents for statistical analysis; counting fingers (CF) at 3 m was converted into 1.85, CF at 2 m to 1.9, CF at 1 m and CF at 30 cm to 2.0, and hand motion to 2.1. Hypotony was defined as an IOP less than 5 mmHg at any postoperative visit, and ocular hypertension was characterized by an IOP higher than 25 mmHg at any visit.

### Surgical technique

Two conjunctival peritomies are created 180° apart nasally and temporally, followed by cauterization in order to achieve hemostasis. A 23-gauge inferior sclerotomy is performed for infusion. Four additional sclerotomies are created: two temporal and two nasal. These sclerotomies are placed 2.5 mm above and below the midline using a trocar blade, at a distance of 3 mm from the limbus to decrease the risk of contact with the iris, permit more reliable centration of the IOL due to the anatomical position being closer to the capsular bag, and decrease the risk of folding of the haptic^9^. A pars plana vitrectomy (PPV) was performed, if needed.

Afterward, the needles of the 7-0 CV-8 Gore-Tex suture (WL Gore & Associates, Newark, DE, USA) are discarded, and the suture thread is cut into two halves. Each end of the thread is then passed through the two adjacent holes in the IOL. The suture is passed anterior-to-posterior and then posterior-to-anterior, a technique described by Fass et al. (2009) as having a reduced risk of iris chafing^14^.

It is important to observe the correct position of the IOL. One of the haptics has an oval opening, and the opposing haptic has a kidney bean-shaped opening. The kidney-bean shaped islet must be positioned in the upper-right quadrant of the patient’s eye.

The anterior chamber can then be accessed through the previously created corneal wound (if cataract surgery was recent) or by constructing a new upper corneal incision using a 2.75-mm blade. The incision can be enlarged to 3.5–4.0 mm.

Each end of the Gore-Tex thread is passed into the anterior chamber and removed from the corresponding sclerotomy using flat intraocular forceps. The Akreos A060 IOL (Bausch & Lomb, Rochester, NY, USA) is folded and placed through the corneal wound into the posterior chamber of the eye, and the four extremities of the thread are then pulled.

Next, the trocars are removed, and the threads are tied. It is important to first center the IOL and then gradually adjust the thread tension at the nasal and temporal extremities before tying the permanent knot. The threads are cut, leaving a long enough tip which, theoretically, may help reduce the risk of future Gore-Tex unfolding and conjunctiva erosion.

In order to avoid postoperative hypotony, leaking sclerotomies can be sutured with 7-0 polyglactin 910 (Vicryl, Ethicon, NJ, USA). The corneal wound is closed using 10-0 nylon suture. Finally, the conjunctival peritomy is also closed, ensuring that the external Gore-Tex thread is completely covered.

### Statistical analysis

Data analysis was performed using the IBM SPSS Statistics software (Statistical Package for Social Sciences, version 23.0), where data frequency and descriptive measures were applied. A *p* value < 0.05 was considered statistically significant.

The Kruskall-Wallis and Mann–Whitney tests were used to compare means and times, and the non-parametric Wilcoxon test was used to verify the existence of significant differences considering paired data.

## Results

A total of 20 patients were included in the current study. Their mean age ± SD at the time of surgery was 64 ± 10 years (range 39–84 years). The study sample included 8 women (40%) and 12 men (60%), and the eyes that underwent surgery were 9 right eyes (45%) and 11 left eyes (55%). Indications for secondary IOL surgery included: dislocated or subluxed crystalline lens after trauma in 7/20 patients (35%); dislocated crystalline lens after complicated cataract surgery in 3/20 (15%); aphakia after complicated cataract surgery in 6/20 (30%) (three of whom had a history of trauma); and dislocated or subluxed IOL in 4/20 (20%) patients (one of these IOL displacements occurred after cataract surgery and another after blunt trauma) (Table 1).

**Table 1:** Baseline patient characteristics.

Characteristics	Data
**Gender, no. (%)**	
Male	12 (60%)
Female	8 (40%)
**Age (years) (mean ± SD)**	64.20 ± 10.75
**Eyes, no. (%)**	
Right eye	9 (45%)
Left eye	11 (55%)
**Surgical indication, no. of eyes (%)**	
Dislocated or subluxed crystalline lens after trauma	7 (35%)
Aphakia after complicated cataract surgery	6 (30%)
Dislocated or subluxed IOL	4 (20%)
Dislocated crystalline lens after complicated cataract surgery	3 (15%)

Ten of the 20 (50%) patients underwent scleral fixation alone, while the other ten eyes underwent concurrent 23-gauge PPV. Of the patients with combined surgery, 4/20 (20%) underwent IOL explantation, 4/20 (20%) lens fragmentation, 1/20 (5%) ERM peeling, and 1/20 (5%) removal of silicone oil and prolene mesh (Table 2). No intraoperative complications were noted.

**Table 2:** Surgical characteristics.

Surgery performed	Data
(1) **Scleral fixation**, no. (%)	**10 (50%)**
** Previous surgery**	
- Complicated cataract surgery	5 (25%)
- PPV + endophacofragmentation	4 (20%)
- PPV + lensectomy + removed IOFB	1 (5%)
(2) **PPV + explant of IOL + scleral fixation**	**4 (20%)**
(3) **PPV + lens fragmentation + scleral fixation**	**4 (20%)**
(4) **PPV + ERM peeling + scleral fixation**	**1 (5%)**
(5) **Removal of silicone oil + scleral fixation**	**1 (5%)**

*ERM*- epiretinal membrane, *IOFB* intraocular foreign body.

### Outcome measures

#### Visual acuity

Mean ± SD uncorrected logMAR visual acuity was 1.92 ± 0.23 (20/1600 Snellen equivalent) preoperatively, which improved significantly to 0.81 ± 0.54, 0.78 ± 0.47, 0.79 ± 0.46, and 0.80 ± 0.56 (20/125 Snellen equivalent) at 1, 2, 3, and 6 months postoperatively, respectively (Wilcoxon-test = − 3.76; *p* value < 0.001).

Mean ± SD best-corrected logMAR visual acuity was 0.43 ± 0.23 (20/50 Snellen equivalent) preoperatively and 0.37 ± 0.24 (20/50 Snellen equivalent) postoperatively (Mann Whitney-test = − 0.66; *p* value = 0.531 and Wilcoxon-test = − 1.01; *p* value = 0.312). BCVA was measured between 3 and 6 months after surgery.

#### Anterior segment changes

On the first day after surgery, 15/20 (75%) patients had corneal edema. After 1 week, this number decreased to 5/20 (25%), at 1 month to 3/20 (15%), and at 2 months to 2/20 (10%). After 3 months of follow-up, none of the patients had corneal edema.

As for anterior chamber cells/flare, 6/20 (30%) were positive for this parameter on the first day, 4/20 (20%) on the seventh postoperative day, and 1/20 (5%) at 1 month and 2 months. At 3 months and 6 months of follow-up, none of the patients was noted to have anterior chamber cells/flare.

A Seidel-positive corneal incision was noted in 1/20 (5%) patient in the first week after surgery. Spontaneous resolution occurred after 2 weeks.

#### Adverse events

**Figure 1:**
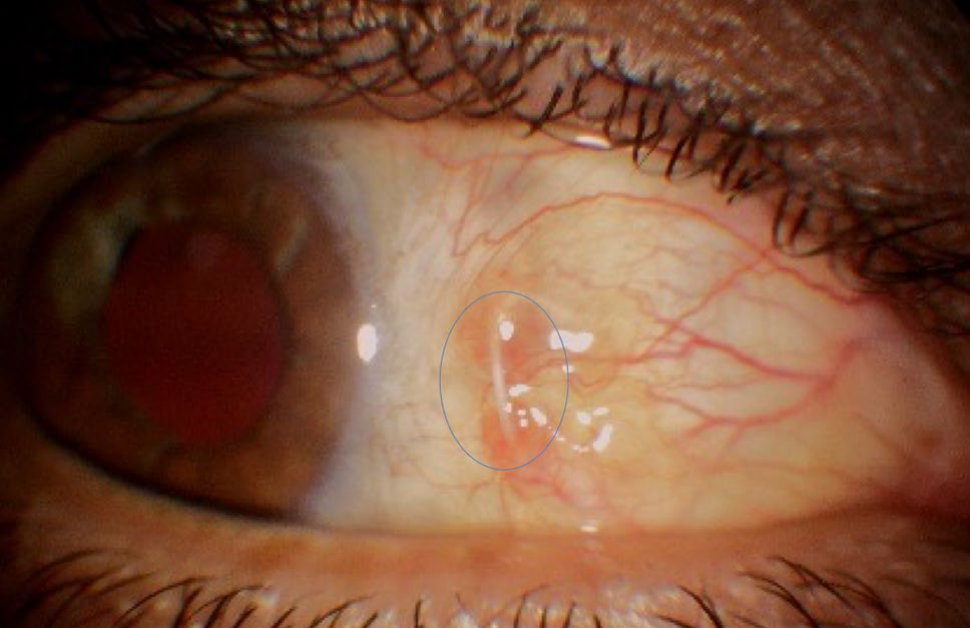
Gore-Tex suture exposure after surgery. In this case, the suture, not the knot, eroded the conjunctiva 7 days after surgery.

The second most common postoperative complication found in the present study, after corneal edema, was conjunctival erosion with exposure of the Gore-Tex suture (Fig. 1); 8/20 (40%) patients exhibited suture exposure during the 6 months of follow-up, decreasing to six cases after conducting procedures on two of them (the exposed tip was cut in one case, and the conjunctiva was resutured in another). Three of the 20 (15%) operated eyes presented with suture exposure in the first week, 4/20 (20%) at 2 months, and 6/20 (30%) at 3 months after surgery.

Iris synechiae were observed in the corneal incision in 4/20 (20%) patients at last follow-up; 2/20 (10%) of them had edema in the upper cornea, but a clear visual axis.

**Figure 2:**
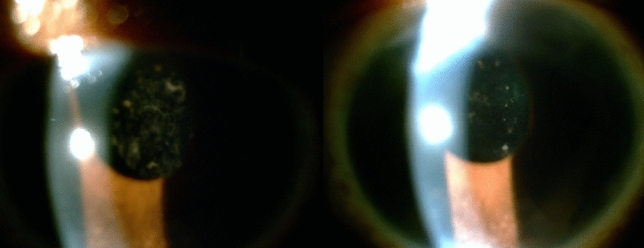
(**a**) Akreos intraocular lens opacification four months after surgery; (**b**) Improvement in lens opacification two months after treatment with topical corticosteroids. The patient had a history of recurrent anterior idiopathic uveitis.

Four months after surgery, 1/20 (5%) patient exhibited anterior chamber cells with opacification of the lens; there was an almost complete improvement in lens opacification and vision after using topical corticosteroids. The patient had a previous history of recurrent anterior idiopathic uveitis (Fig. 2a,b).

On the first postoperative day, 1/20 (5%) patient presented with macular intraretinal hemorrhage. OCT was performed 4 months postoperatively after refraction (BCVA was 20/200) and demonstrated disorganization of the outer retina. One case of probable toxic anterior segment syndrome (TASS) presented on the first postoperative day. Due to the possibility of endophthalmitis, intravitreal antibiotic injection was performed, and the patient achieved a final BCVA of 20/40. Three patients (3/20; 15%) developed cystoid macular edema (CME) and 2/20 (10%) developed epiretinal membrane (ERM); both pathologies were noted after 6 months of follow-up.

Two patients (2/20; 10%) had transient vitreous hemorrhages (resolution within 1 month), while 1/20 (5%) presented with recurrence of a retinal detachment (RD) (the patient had had a RD about 10 years prior), which was observed 20 days after placement of the secondary IOL. The recurrent RD was repaired with a pneumatic retinopexy with laser photocoagulation. Corneal de-epithelialization was observed after the laser and herpetic keratitis developed a few weeks later. After treatment, the patient developed a central corneal opacity and final BCVA was 2.1 logMAR.

### Specular microscopy

Mean ± SD endothelial count was 1740.50 ± 522.92 cells/mm^2^ (range 1019.00–2849.00) preoperatively and 1187.19 ± 493.00 cells/mm^2^ (range 657.00–2262.00) postoperatively (Mann Whitney-test = − 3.18; *p* = 0.001 and Wilcoxon-test = − 3.52; *p* < 0.001). Specular microscopy and pachymetry were performed at baseline and 3 to 6 months after surgery.

Mean ± SD pachymetry was 562.00 ± 62.07 µm (range 439.00–687.00) preoperatively and 564.65 ± 65.71 µm (range 450.00–741.00) postoperatively (Mann Whitney-test = − 0.30; *p* = 0.775 and Wilcoxon-test = − 0.45; *p* = 0.653). Due to such factors as corneal opacity, the endothelial cell count and pachymetry could not be performed after surgery in 4 of the 20 patients.

### Intraocular pressure (IOP) and IOP-lowering therapy

Mean ± SD IOP was 14.95 ± 3.14 mmHg at baseline and was 11.40 ± 5.64 mmHg on postoperative day 1, 14.25 ± 5.31 mmHg at 1 week after surgery, 15.10 ± 4.48 mmHg at 1 month, 14.75 ± 5.96 mmHg at 2 months, 15.35 ± 5.09 mmHg at 3 months, and 15.15 ± 4.06 mmHg at 6 months after surgery (Kruskall Wallis-test: *p* = 0.493).

Hypotony (IOP < 5 mmHg) was observed in 3/20 (15%) patients on the first postoperative day. After one week, all three patients had normal IOP, and no complications of ocular hypotony were identified.

Preoperatively, none of the patients had IOP > 25 mmHg, and 3/20 (15%) were using IOP-lowering medication. Over the course of the 6-month follow-up, 2/20 (10%) patients exhibited ocular hypertension. At the 6-month evaluation, none of them had an IOP > 25 mmHg, and 5/20 (25%) were using IOP-lowering agents. Nine of the patients (9/20; 45%) showed an IOP increase of at least 5 mmHg throughout the follow-up compared to baseline.

### Refractive changes

In the pre and postoperative periods, respectively, the mean ± SD sphere power in diopters was + 11.76 ± 3.00D (range + 1.25 to + 15.00) and + 0.46 ± 1.34D (range − 1.50 to + 3.0) (Mann Whitney-test = − 5.25; *p* < 0.001); the mean ± SD cylinder power was − 1.32 ± 1.60D (range − 6.0 to 0) and − 3.21 ± 1.57D (range − 1.0 to − 6.0) (Mann Whitney-test = − 3.61; *p* < 0.001); and the mean ± SD spherical equivalent was + 11.06 ± 3.06D (range + 0.25 to + 14.75) and − 1.12 ± 1.50D (range − 4.25 to + 1.0) (Mann Whitney-test = − 5.25; *p* < 0.001).

## Discussion

New scleral fixation techniques have recently been described using alternative sutures, foldable lenses, and smaller caliber instruments^1,6,15–22^. Retrospective studies have shown favorable results of scleral fixation using the Akreos AO60 IOL and Gore-Tex suture^5,7,9–13^. To our knowledge and based on a computerized search of the Medline database, this is the first prospective study of Akreos A060 scleral fixation with Gore-Tex suture, and it demonstrates that the technique is generally safe and associated with favorable visual results.

Fixing an IOL at two points can cause IOL tilt or decentration^23^. The Akreos AO60 design facilitates centration and decreases tilt, thus reducing induced astigmatism and high-order aberrations. Also, it is easily foldable, allowing a smaller corneal incision, which maintains the stability of the anterior chamber and prevents intraoperative hypotony. Moreover, since it is an aspherical lens, free from aberrations, the Akreos AO60 can provide superior quality vision which is not affected by decentration or pupil size^8^. This constitutes an additional advantage of the Akreos AO60 in eyes with traumatic mydriasis, a relatively common condition in patients undergoing scleral fixation of an IOL. Also, because it is hydrophilic acrylic, it is associated with less inflammation when compared to hydrophobic acrylic lenses^24^.

As for the type of suture, prolene has traditionally been the most frequently used thread for scleral fixation. However, due to complications related to the thread, Gore-Tex suture is being used more and more for this procedure, as it provides advantages such as greater resistance to traction and small memory, which facilitates manipulation and causes a minimal inflammatory response^21^.

**Figure 3:**
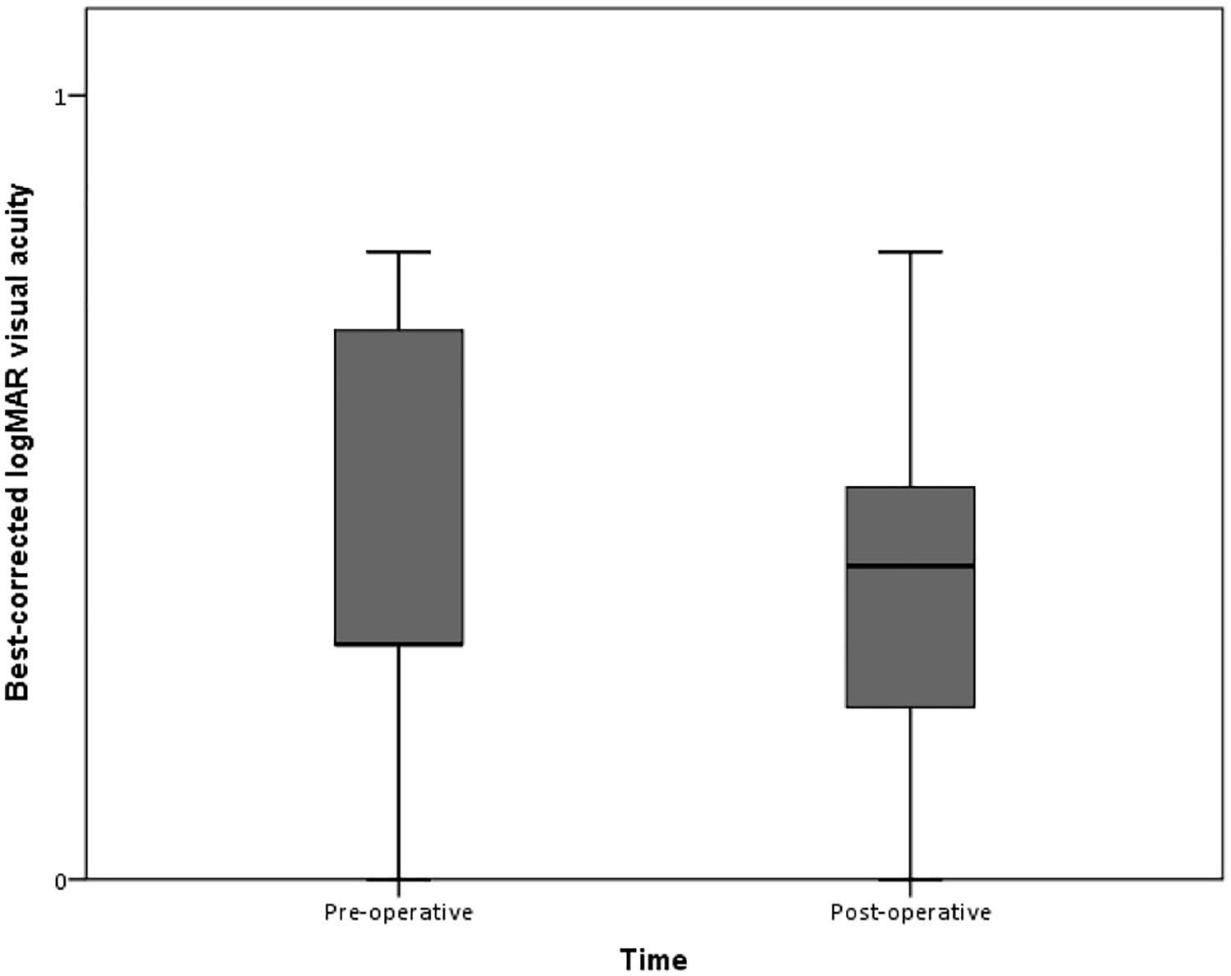
Box plot graphics show the similarity in best-corrected visual acuity (BCVA) at baseline (preoperative: mean logMAR BCVA: 0.43) and 3–6 months after surgery (postoperative: mean logMAR BCVA: 0.37). Middle line represents the median, the square represents the mean, the 25th and 75th percentiles determine the box, and the 5th and 95th percentiles determine the whiskers.

**Figure 4:**
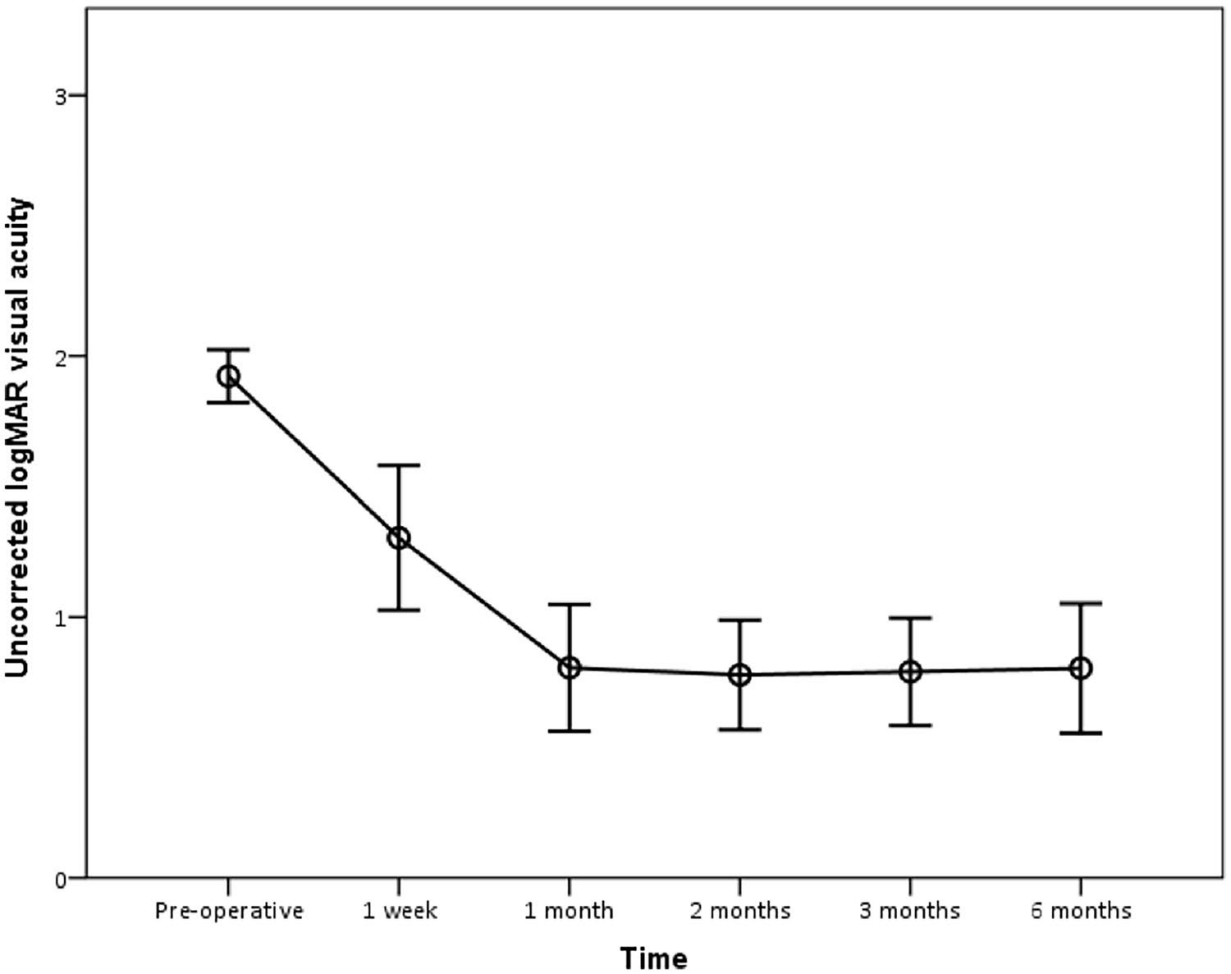
The mean uncorrected logMAR visual acuity pre and postoperatively (1.92 preoperative, 1.30 at postoperative week 1, 0.81 at postoperative month 1, 0.78, 0.79 and 0.8 at postoperative months 2, 3 and 6). There was significant improvement in uncorrected visual acuity after IOL scleral fixation at all study visits when compared to baseline (*p* ≤ 0.001). Mean visual acuity was stable between postoperative months 1 and 6.

In the present study, all patients were followed-up for at least 6 months. One of the most important parameters to assess the safety of the technique is visual acuity. We observed a BCVA that was very similar to the preoperative value (0.43 at baseline and 0.37 after surgery; *p* = 0.531) (Fig. 3). Malamud et al. (2016)^20^ and Khan et al. (2019)^5^ reported outcomes in series of 57 and 63 eyes, respectively, undergoing PPV with ACIOL or scleral-sutured PCIOL placement; they obtained similar visual results to those in our study: 20/60 (ACIOL group) and 20/40 (CZ70BD group) in Melamud et al.; 20/50 (ACIOL group) and 20/46 (Akreos AO60 group) in Khan et al. Importantly, Khan et al. (2019)^5^ and Melamud et al. (2016)^20^ stated that eyes with significant ocular history or visually significant macular pathologies were excluded. Further, 18/20 (90%) of our patients exhibited improvements in visual acuity when comparing the uncorrected visual acuity before and after surgery and 14/20 (70%) had equal or better final visual acuity when comparing the pre and postoperative corrected acuities. Visual stability was achieved after the first month (Fig. 4).

In this study, corneal edema was the most frequent complication in the immediate postoperative period (75% on day 1). Khan et al. (2019)^5^ reported transient corneal edema (less than 1 month) in 12 (19%) eyes; Patel et al. (2018)^21^ found significant persistent corneal edema (longer than 1 week) in 4/49 (8.2%), and one patient with a predisposition to endothelial disease developed prolonged edema. We observed a statistically significant variation in the endothelial cell count among our patients (mean of 1740.50 cells/mm^2^ preoperatively to 1187.19 cells/mm^2^ postoperatively; *p* < 0.001). Specular microscopy data are not routinely discussed in previous series of scleral fixation, even though such information constitutes an important factor related to the risk of corneal decompensation. None of the patients in our study developed chronic corneal edema, but all of our patients had an endothelial cell count above 1000 cells/mm^2^ at baseline. In 2017, Yamane et al. noted that the mean corneal endothelial cell density (ECD) decreased from 2341 to 2313 cells/mm^2^ and 2244 cells/mm^2^ at 6 and 36 months, respectively^25^. This small variation can be explained by the different technique and higher initial ECD, which makes it less susceptible to endothelial cell loss. Despite the reduction in the endothelial cell count between pre and postoperative periods, there was no significant change in the pachymetry values (mean of 562 µm preoperatively to 564 µm postoperatively; *p* = 0.775), consistent with the absence of any cases of chronic edema.

The second most common postoperative complication in the present study was conjunctival erosion with suture exposure; 8/20 (40%) patients exhibited Gore-Tex exposure throughout the 6-month follow-up period. Rotating the suture may warp this soft lens, especially if the suture is too tight^27^. We chose not to rotate and bury the suture due to the risk of displacing the one-piece IOL, a fact that may have led to the higher rate of suture exposure in our study compared to in other studies. Due to the high incidence of suture erosion that we observed, we recommend rotating and burying the knots after tightening them with adequate tension or burying the knot under a scleral flap, as reported by Fass et al. (2010)^15^. Goel et al. (2018)^11^ stated that the use of scleral flaps and the rotation of suture knots decrease the risk of suture erosion. In their 2015^7^ and 2018^9^ publications, Khan et al. did not observe any suture-related complications, including rupture, erosion, IOL dislocation or tilt, endophthalmitis, or persistent postoperative inflammation. In 2020, Bonnell et al.^22^ reported suture exposure in 1/15 (7%) patients, and in 2018 Patel et al.^21^ reported one patient with Gore-Tex suture erosion with purulent scleritis, even though the knot was rotated and buried.

Another important consideration is anterior segment inflammation. We observed that the presence of cells in the anterior chamber was an uncommon event (20% of patients on the seventh postoperative day), and had a maximum duration of 3 months. This information is not usually mentioned in studies on scleral fixation with Akreos AO60, although it is relevant since, theoretically, this hydrophilic acrylic lens causes less inflammation than hydrophobic acrylic lenses and silicone lenses^24^. Khan et al. (2016)^7^ reported a range between 1.1% and 5.4% of anterior uveitis associated with iris fixation, scleral fixation, and anterior chamber IOLs noted in other studies.

As for lens-related complications, one patient presented with cells in the anterior chamber and lens opacification in the fourth month after surgery. Opacification of the Akreos AO60 and other such hydrophilic IOLs has been reported in the literature, including in diabetic patients and in patients with a history of anterior uveitis, and associated with gas tamponade^26,27^; therefore, some surgeons have changed their preferred IOL to hydrophobic acrylic IOLs, but unfortunately there is no hydrophobic 4-point IOL available in the Brazilian market. In our case, no gas was used intraoperatively, and there was an improvement in the anterior chamber inflammation and lens opacification after using topical corticosteroids. Of note, the patient in our study who presented with anterior chamber cells and lens opacification had a history of recurrent anterior idiopathic uveitis.

Two (10%) patients in our study had transient vitreous hemorrhages (VH), with resolution within 1 month. The VH were probably related to scleral wound sutures and hypotony at the end of surgery. Three of the 20 (15%) eyes presented with hypotony (IOP < 5 mmHg) on the first postoperative day, without secondary complications. This may have been caused by leakage of the sclerotomies, which were sutured at the end of the procedure only when necessary. At 1 week after surgery, all IOPs had normalized, probably due to sclerotomy wound healing. Methods to decrease the risk of postoperative hypotony include performing a fluid-air exchange at the end of surgery (although this increases the risk of lens opacification), using smaller gauge instruments and partially fill the anterior chamber with viscoelastic. In 2014, Khan et al.^1^ observed transient hypotony in 9.4% of their patients, but in their 2018 study there were no cases of hypotony; 23-gauge and 23 or 25-gauge instruments were used, respectively. In one of these two cases of vitreous hemorrhage, retinal detachment (RD) was observed 20 days after the surgery. Following pneumatic retinopexy, the retina was successfully attached, but after a laser session, the patient developed corneal de-epithelialization, herpetic keratitis, and corneal opacity (2.1 logMAR final acuity). The incidence of VH ranges from 0 to 12.2% in studies reporting other IOL implantation techniques^7^.

After 6 months of follow-up, 2 (10%) of our patients were noted to have ERM and 3 (15%) had CME. In the current study, since OCT was not routinely performed in all patients, it is not possible to determine with certainty when during the postoperative period the ERM and CME developed, or whether these retinal findings were already present prior to surgery. Preoperative OCT was not possible due to media opacity, such as retained lens material. In 2017, Khan et al. reported CME in 4.8% of patients; they observed this complication in patients who underwent pars plana lensectomy concomitantly with phacofragmentation for traumatic cataracts, subluxed crystalline, or retained lens material.

Throughout the 6-month follow-up in our study, 2/20 (10%) patients presented with ocular hypertension (Table 3), and at the 6-month evaluation, none of the patients had IOP ≥ 25 mmHg. Ocular hypertension was a finding described in all recent studies of scleral fixation with sutures, with the reported proportion of affected patients being 3.6%^9^, 7%^1^, 7.9%^5^, 16.3%^21^, and 24%^10^. The proportion of patients using IOP-lowering agents and IOP variation comprise two other important considerations. Twenty five percent of our patients were taking IOP-lowering eye drops after 6 months of follow-up, despite having normalized IOPs, and 45% showed an IOP increase of at least 5 mmHg over the course of follow-up when compared to baseline.

**Table 3:** Postoperative complications.

Postoperative complications	No. of eyes (%)	Date of the first and last observation of the occurrence	Therapy/outcome
Corneal edema	15 (75%)	1 day–60 days	Observation/resolution
Exposure of suture	8 (40%)	7 days–6 months	–
ACR	6 (30%)	1 day–60 days	Observation/resolution
Ocular hypotony	3 (15%)	1 day	Observation/resolution
CME	3 (15%)	After 6 months	In treatment
ERM	2 (10%)	After 6 months	–
Vitreous hemorrhage	2 (10%)	1 day–7 days	Observation/resolution
Ocular hypertension	2 (10%)	1 week–3 months	Topical hypotensive drugs
Opacification of IOL	1 (5%)	120–150 days	Topical corticosteroids
TASS	1 (5%)	1 day–7 days	ATB + Dexa IV
Retinal detachment	1 (5%)	21–30 days	C3F8 + laser/resolution

*ACR* anterior chamber reaction, *CME* cystoid macular edema, *ERM* epiretinal membrane, *TASS* toxic anterior segment syndrome.

In spite of the safety and favorable visual results associated with scleral-fixated IOL implantation, little is known regarding the refractive outcomes associated with scleral-fixated IOLs and the optimal IOL calculation formula for use with these lenses^6^. In 2016, Terveen et al.^10^ reported refractive results from 18 eyes which underwent scleral fixation of Akreos A060 with 9–0 Prolene or Gore-Tex. The mean postoperative spherical power was + 0.08 ± 1.31D (SD) (range − 2.75 to + 2.00 D), which is comparable to the mean postoperative spherical power in our study (+ 0.46 ± 1.34D [SD] [range − 1.50 to + 3.0]). In the present study, the mean postoperative spherical equivalent (SE) ± SD and the mean residual SE (postoperative SE minus target refraction) were − 1.12 ± 1.50D and − 0.72D, respectively, values that are similar to those reported by Botsford et al.^12^ (− 0.79 ± 0.95D and − 0.19D as mean postoperative SE ± SD and mean residual SE, respectively) among 31 patients who underwent scleral fixation of Akreos A060 or CZ70BD lenses with CV-8 Gore-Tex suture placed 3 mm posterior to the limbus.

In 2019, Su et al.^13^ reported a mean surgically induced astigmatism (SIA) of 0.77D, a much lower value than that found herein, which was − 3.21 ± 1.57D (range − 1.0 to 6.0). It is noteworthy that, in general, reported astigmatism is lower in more recent studies, such as that by Su et al. (2019)^13^. Since the new lenses, such as Akreos A060, are foldable, it is possible to make smaller corneal incisions. However, incision expansion may be required for IOL explantation. In the present study, 6 patients underwent IOL explantation, a procedure that has declined in frequency due to the availability of improved instrumentation, such as scissors to cut the lens^26^. We also observed a greater postoperative astigmatism after complicated cataract surgery. Theoretically, making scleral tunnels can lead to less induced astigmatism, and may be an alternative in patients in whom a new incision is needed, as in cases in which it is not possible to use the corneal incision that was made in previous surgery. Beyond astigmatism induced by the corneal incision, we also attribute the high astigmatism to the possibility that the lens was sutured too tight in some cases, despite the care taken. As mentioned above, this could have hindered rotation of the knot. We reaffirm the importance of adjusting the tension of the suture before tying the knot.

Limitations of the present study include its limited sample size and relatively short duration of follow-up. Longer-term follow-up is warranted to evaluate such potential complications as suture breakage and IOL dislocation. Another limitation of this study is that other surgical techniques were not evaluated, thus hampering comparison. Also, pre and postoperative keratometry were not analyzed for comparison and to define corneal astigmatism. However, we observed greater total astigmatism in patients who had an IOL explant. Further, the rates of postoperative complications and the final visual acuity may have been influenced by posterior segment pathology. It would have been interesting to perform OCT before and after surgery in all patients, throughout follow-up, to identify retinal changes and correlate them with visual acuity. However, preoperative OCT was not possible due to media opacity, such as retained lens fragments.
